# Could Peer Support Programs Be a Good Resource for Managing the Unmet Needs of Cancer Patients?

**DOI:** 10.1007/s13187-018-1399-4

**Published:** 2018-08-08

**Authors:** Hwa Yeon Park, Mi Jin Kim, Ju Young Kim, Sarah Kim, Ji Young Choi, Jeong Hyun Kim, Hee Yeong Jeong

**Affiliations:** 1 Health Promotion Center, Gachon University Gil Medical Center, 21 Namdong-daero 774beon-gil, Namdong-gu, Incheon, Republic of Korea; 2 College of Nursing, Shin Han University, 30 Beolmadeul-ro 40beon-gil, Dongducheon-si, Gyeonggi-do Republic of Korea; 3 Department of Family Medicine, Seoul National University Bundang Hospital, 82 Gumi-ro 173beon-gil, Bundang-gu, Seongnam-si, Gyeonggi-do Republic of Korea; 4 International Healthcare Center, Seoul National University Bundang Hospital, 82 Gumi-ro 173beon-gil, Bundang-gu, Seongnam-si, Gyeonggi-do Republic of Korea; 5 Center for Cancer Information and Education, Seoul National University Bundang Hospital, 82 Gumi-ro 173beon-gil, Bundang-gu, Seongnam-si, Gyeonggi-do Republic of Korea; 6 Department of Psychiatry, Seoul National University Bundang Hospital, 82 Gumi-ro 173beon-gil, Bundang-gu, Seongnam-si, Gyeonggi-do Republic of Korea; 7 Health Promotion Center, Seoul National University Bundang Hospital, 82 Gumi-ro 173beon-gil, Bundang-gu, Seongnam-si, Gyeonggi-do Republic of Korea

**Keywords:** Cancer survivorships, Unmet needs, Peer support needs, Mentorship, Peer support programs

## Abstract

The number of cancer patients has been rapidly increasing, and while there have been wide variations, cancer survival rates also improved globally. Despite the improved survival rates, supportive care needs of cancer patients have been unmet in various domains. The current study aimed to investigate unmet needs that had potential to be managed by peer supports according to cancer trajectories. We used the comprehensive needs assessment tool in cancer (CNAT) and a modified CNAT to evaluate the unmet needs and peer support needs of cancer patients at the tertiary hospital of South Korea. Of the 402 participants, 335 (83.3%) needed peer support. For patients who had been diagnosed with cancer for more than 5 years, the highest proportion of peer support needs to unmet supportive care was reported in information domain (92.9%). Patients with advanced cancer reported peer support needs in the social/religious/spiritual (84.4%) and practical domains (81.1%). Most of stomach cancer patients needed peer supports to receive information (96.6%). The need for peer supports in the information domain was reported highest according to longer survival period and also according to advanced cancer stages. The proportion of peer support needs in unmet supportive care varied by cancer type. Further interventional studies are needed to investigate satisfaction with peer support in specific domains.

**Electronic supplementary material:**

The online version of this article (10.1007/s13187-018-1399-4) contains supplementary material, which is available to authorized users.

## Introduction

The number of cancer patients has been rapidly increasing worldwide as a result of the aging population and the improvements in early diagnosis and treatment. It has been estimated that there are 32.6 million 5-year cancer survivors and 14 million new cancer cases worldwide each year [1], and the number of new cases is expected to increase by approximately 70% over the next two decades [2]. Though there have been wide variations, globally, cancer survival has slightly improved [3]. Despite the improved survival rates, cancer patients’ specific medical, psychological, informational, or social needs have reported to be largely unmet [4].

Previous studies addressing unmet needs of cancer patients mostly focused on patients with cancer stages, such as early or advanced, or with specific time periods, such as newly diagnosed cancer or long-term survivorship [5–8]. Cancer survivorship was described as time periods of survival with three phases-acute, extended, and permanent survivorship [9]. Although there are no distinctive time boundaries between the phases, patients confront to common challenges through the phases from the moment they were diagnosed with cancer to free-of-disease status [10]. Therefore, it is essential to assess unmet supportive care needs of cancer patients and examine possible resource of support according to cancer trajectories.

In this manner, social support including peer support can be a good resource in overcoming stress and has been positively associated with post-traumatic growth [11] as well as improvements in the quality of life of cancer patients [12]. One study examining the sources of supportive care in breast cancer illustrated a high need for peer support in addressing medical-psychological, social-spiritual, and sexual needs [13].

Peer support could be defined as being helped by a group of people who have the same disease and gathered together to exchange information, shared experiences, or give encouragement to each other to overcome the disease. Peer support programs which consist of cancer survivors as mentors furnish cancer patients with unique emotional and educational benefits [14]; furthermore, they represent a low cost approach and enable reallocation of some aspects of cancer care to community settings [15]. Targeting the right patients in their time of need by delivering timely informational as well as psychosocial support is essential to maximize the effect of peer support as a positive resource.

However, several review articles that explored the benefit of peer support program showed mixed results and potential mediating or moderating socio-economic factors (e.g., marital status, educational status, or economic status) on psychological outcomes [16, 17]. Also, a detailed description regarding the peers who engaged in the one-to-one support program or recruitment process seemed limited and there might have had potential conflicts with traditional professional-based care and peer-supported program. Cost-effectiveness issues should be considered when managing the peer support program because of staff time for recruiting and supervising, development of delivery methods for peer support, or educating the support providers appropriate knowledge regarding cancer trajectory.

Unmet supportive needs can vary according to the time of cancer diagnosis [4, 18]. At the cancer diagnosis phase, anxiety with fears was commonly identified, and during treatment phase, most unmet needs were highly reported in all domains. As time went by, many cancer patients reported persistent unmet care needs in several domains. Although unmet supportive care needs are prevalent among cancer patients, effective interventions to reduce unmet needs have been limited [19]. Though peer support might have some potential in fulfilling these unmet care needs, few studies have matched peer support domains with the highly unmet needs of cancer patients according to the stage of their cancer trajectory.

Therefore, as a basis of designing peer support programs, the aim of this study was to (1) identify unmet supportive care needs of cancer patients and (2) determine the domains for which patients considered peer support a good resource in terms of time since cancer diagnosis.

## Methods

### Study Designs and Participants

This cross-sectional study was conducted from January 2014 to April 2014 in a comprehensive cancer center of a tertiary hospital in South Korea. Around 600 patients were approached, 47 refused to participate and 50 did not meet inclusion criteria or exclusion criteria, so a total 503 patients were recruited. Inclusion critieria were (1) over 19 years old, (2) with diagnosis of cancer, and (3) currently undergoing evaluation for cancer stage or treatment stage or post-treatment follow-up period. Patients who had an Eastern Cooperative Oncology Group Performance Status (ECOG PS) score of 3 (capable of only limited self-care) to 5 (dead) [20] or difficulty in understanding the questionnaire were excluded.

A trained nurse conducted face-to-face interviews with visiting patients in outpatient waiting room of cancer clinics, inpatients wards, or cancer information and education center at the tertiary hospital. She explained the purpose of study and, after receiving written informed consent, asked participants to complete a questionnaire. The survey conducted in three steps. First, we assessed supportive care needs of all cancer patients using comprehensive needs assessment tool in cancer (CNAT) [21]. Second, we explained the meaning of peer support, then asked the patients if they need peer support. Third, for those who responded “yes,” we examined peer support needs with modified CNAT.

To explore the unmet needs and peer support needs of cancer patients in relation to the duration of their survival, we divided them into four groups according to the time since cancer diagnosis. These groups were determined based on patients’ treatment and follow-up schedules: acute survivorship, less than 3 months; transitional survivorship, 3 months–2 years; extended and chronic survivorship, 2–5 years; and permanent survivorship, more than 5 years.

### Measures

#### Supportive Care Needs

We used CNAT to examine supportive care needs. The CNAT is self-administered questionnaire, which comprehensively measures cancer patients’ needs and has been developed to cover various cancer types and all phases of cancer experiences after diagnosis. It consists of 59 items with seven domains: information (10 items), psychological care (10 items), health care staff (8 items), physical symptoms (12 items), hospital facilities and services (8 items), social/religious/spiritual support (6 items), and practical support (6 items). Internal consistency of the CNAT was reported satisfactory (Cronbach’s alpha = 0.97 for the total scales) [21].

#### Peer Support Needs

We described peer support as being helped by a group of people who have the same disease and gather together to exchange information, share experiences or give encouragement to each other to overcome the diseases.

To assess peer support needs, we modified some of the questions in the CNAT on basis of clinical knowledge, context of questionnaire, cultural context, and expert opinions including psychiatrist, oncologist, and oncology nurse practitioners. Through the meeting with experts, some items about unmet needs from health care staff or hospital facilities and services that could not be replaced by peer support were removed. Besides these items, a phrase of “peer support” was inserted in the questions.

For example, the items “Wished to see doctor in a quick and easy way when in need,” “Wished my nurse to promptly attend to my discomfort and pain,” or “Wished to have short waiting period between the reservation and the doctor’s appointment” were removed. The item “Needed information or education about things that I can do at home for my health” was changed to “Needed peer support to receive information or education about things that I can do at home for my health.” Another item such as “Needed help with feelings of anger, irritability, or nervousness” was also changed to “Needed peer support with feelings of anger, irritability, or nervousness.”

By deleting or rephrasing items, modified CNAT comprised 40 items. The questions regarding the need for peer support consisted of five domains: information (10 items), psychological care (10 items), physical symptoms (12 items), social/religious/spiritual support (4 items), and practical support (4 items).

Patients were asked to specify their level of unmet needs or their level of needs for peer support regard to the past month using a four-point Likert scale (0 = no need, 1 = low need, 2 = moderate need, 3 = high need). For each item, a score was given 0, if patients had no difficulties or if the item was not relevant. If patients had needs for each question, they marked the score from 1 to 3 depending on level of need.

Since our main aim was to explore the potential utilization of peer support program in moderate-to-high level of unmet needs in cancer patients, we defined having unmet needs as at least having moderate to high needs (Likert scale more than 2). Patients who reported moderate-to-high need at least one item in a domain was considered to have needs in that domain. Also, if patients were eligible and asked to complete modified CNAT for need of peer support, same held true to have peer support needs in that domain.

### Statistical Analysis

Descriptive statistics were used to describe the socio-demographic characteristics and the 10 items with the highest unmet need. Chi-squared test and Fisher’s exact tests were used for categorical variables.

To assess how unmet needs and peer support needs matched, dichotomous variables were created as having moderate-to-high unmet needs (yes/no) and having moderate-to-high peer support needs (yes/no). We calculated the ratio of peer support needs compared with unmet needs in each domain across the type, duration, or stage of cancer and compared the ratio using independent two population proportion test. All tests of significance were two-sided, and a *p* value < 0.05 was considered statistically significant. All analyses were performed using STATA version 14.0 (Stata Corp., TX, USA).

## Results

### Participant Characteristics

Of the 503 eligible participants, 402 completed the questionnaire. The participants’ median age was 58 years (range 22–89 years). More than half of the subjects were women (66.4%), and most were married (86.0%), and had primary cancer without recurrence (85.1%) when the survey was conducted. Approximately a third of participants (36.1%) were diagnosed with cancer 3 months to 2 years ago. The most common cancer was breast cancer (36.8%), followed by colon/rectum cancer (18.7%), thyroid cancer (11.0%), stomach cancer (9.0%), prostate cancer (5.5%), and lung cancer (4.7%). Additionally, 87.1% of subjects had received surgery to treat cancer, 61.4% chemotherapy, and 29.1% radiation therapy, with multiple answers allowed (Table 1).

**Table 1:** Participant characteristics (*N* = 402)

	Number	Percent
Age, median (range)	58 (22–89)	
Age group		
≤ 39 years	41	10.2
40–59 years	177	43.9
≥ 60 years	184	45.8
Gender		
Men	135	33.6
Women	267	66.4
Education^a^		
≤ High school	229	61.9
≥ College	141	38.1
Marital status^a^		
Married	318	86.0
Separate/divorced/widowed	27	7.3
Never married	25	6.8
Cancer stage		
1	120	29.9
2	141	35.1
3	97	24.1
4	44	11.0
Time since cancer diagnosis		
< 3 months	68	16.9
3 months–2 years	146	36.3
2–5 years	113	28.1
≥ 5 years	75	18.7
Cancer type		
Breast	148	36.8
Colon/rectum	75	18.7
Lung	19	4.7
Thyroid	44	11.0
Stomach	36	9.0
Prostate	22	5.5
Others^b^	58	14.4
Currently undergoing treatment		
Yes	169	42.0
No	233	58.0
Recurrence		
Yes	60	14.9
No	342	85.1
Treatment^c^		
Surgery	350	87.1
Chemotherapy	247	61.4
Radiation	117	29.1

^a^Excluded missing data

^b^Included esophagus, kidney, liver, pancreas, prostate, ovary, cervix, leukemia, and lymphoma

^c^Multiple answers were allowed

### Description of the Most Common Unmet Needs of Cancer Patients

Of the 402 participants, 381 (94.8%) reported at least one unmet need in overall domains. The most frequently reported unmet needs in cancer patients were related to information and education. The highest unmet need among cancer patients was for information on complementary and alternative medicine (69.4%) and education about things that patients can do at home (69.4%). Other unmet needs in cancer patients were reported in the following order: “correct diet-which foods to eat and to avoid” (68.2%), “information about financial support for medical expenses from government” (67.9%), “rehabilitation medical services to help with functional recovery after treatment (67.7%), and “my doctor to be easy, specific, and honest in his/her explanation” (66.9%).

### Prevalence of Unmet Needs by Supportive Care Needs Domains

Table 2 shows the prevalence of at least one moderate-to-high unmet need reported by patients according to sociodemographic and clinical factors. Cancer patients had the most unmet needs in the information domain (88.6%) and the least in the social/religious/spiritual support domain (69.4%).

**Table 2:** Moderate to high unmet needs by supportive care needs domain

	Information		Psychological care		Health care staff		Physical symptoms		Hospital facilities and services		Social/religious/spiritual support		Practical support	
	*n*	%	*n*	%	*n*	%	*n*	%	*n*	%	*n*	%	*n*	%
Total	356	88.6	299	74.4	308	76.6	310	77.1	326	81.1	279	69.4	292	72.6
Cancer stages^a^														
1	104	86.7	80	66.7	79	65.8	80	66.7	87	72.5	70	58.3	81	67.5
2	126	89.4	106	75.2	112	79.4	111	78.7	117	83.0	99	70.2	104	73.8
3	85	87.6	77	79.4	81	83.5	82	84.5	84	86.6	76	78.4	73	75.3
4	41	93.2	36	81.8	36	81.8	37	84.1	38	86.4	34	77.3	34	77.3
Period														
< 3 months	65	95.6	55	80.9	54	79.4	55	80.9	56	82.4	51	75.0	50	73.5
3 months–2 years	126	86.3	105	71.9	109	74.7	107	73.3	112	76.7	99	67.8	100	68.5
2–5 years	99	87.6	85	75.2	86	76.1	90	79.6	94	83.2	75	66.4	84	74.3
≥ 5 years	66	88.0	54	72.0	59	78.7	59	78.7	64	85.3	54	72.0	58	77.3
Cancer types^b^														
Breast	130	87.8	99	66.9	100	67.6	102	68.9	105	70.9	90	60.8	98	66.2
Colon/rectum	62	82.7	51	68.0	55	73.3	55	73.3	61	81.3	46	61.3	50	66.7
Lung	16	84.2	15	78.9	15	78.9	14	73.7	17	89.5	14	73.7	12	63.2
Thyroid	42	95.5	40	90.9	40	90.9	40	90.9	40	90.9	40	90.9	39	88.6
Stomach	32	88.9	29	80.6	31	86.1	30	83.3	31	86.1	29	80.6	29	80.6
Prostate	18	81.8	15	68.2	17	77.3	17	77.3	18	81.8	17	77.3	16	72.7
Others^c^	56	96.6	50	86.2	50	86.2	52	89.7	54	93.1	43	74.1	48	82.8
Recurrence^d^														
Yes	56	93.3	51	85.0	51	85.0	55	91.7	56	93.3	51	85.0	50	83.3
No	300	87.5	248	72.3	257	74.9	255	74.3	270	78.7	228	66.5	242	70.6

^a^Statistically significant in health care staff (*p* < 0.01), physical symptoms (*p* < 0.01), hospital facilities and services (*p* < 0.05), and social/religious/spiritual support domains (*p* < 0.01)

^b^Statistically significant in psychological care (*p* < 0.01), health care staff (*p* < 0.01), physical symptoms (*p* < 0.01), hospital facilities and services (*p* < 0.01), social/religious/spiritual support (*p* < 0.01), and practical support domains (*p* < 0.05)

^c^Included esophagus, kidney, liver, pancreas, prostate, ovary, cervix, leukemia, and lymphoma

^d^Statistically significant in psychological care, physical symptoms, hospital facilities and services, social/religious/spiritual support, and practical support domains (all *p* < 0.05)

Patients with more advanced stages of cancer had current needs in health care staff (*p* = 0.009), physical symptoms (*p* = 0.008), hospital facilities and service (*p* = 0.03), and social/religious/spiritual support (*p* = 0.008). Among cancer types, patients have significant different unmet needs in all domains except for information domain, and 90% of thyroid cancer patients were likely to have most unmet needs. Cancer patients with recurrent cancer reported statistically significant higher needs in psychological care (*p* = 0.004), physical symptoms (*p* = 0.004), hospital facilities and service (*p* = 0.009), social/religious/spiritual support (*p* = 0.004), and practical support domains (*p* = 0.04).

### Proportion of Peer Support Needs to Unmet Needs in Cancer Patients

**Fig. 1:**
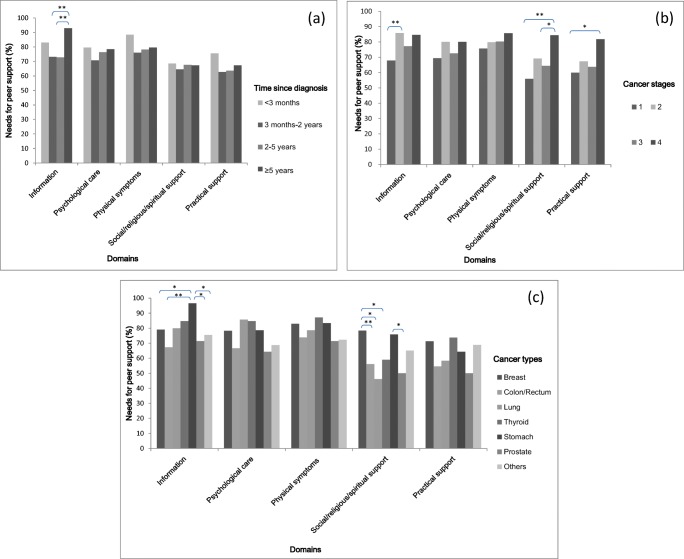
Proportion of peer support needs to unmet needs according to time since cancer diagnosis (**a**), cancer stages (**b**), and cancer types (**c**). *p < 0.05, **p < 0.01

Of the 402 participants, 335 (83.3%) needed peer support. The highest proportion of patients with peer support needs to unmet needs was in those who had been diagnosed with cancer for more than 5 years in the information domain, at 92.9%. This proportion differed significantly from that of participants 3 months–2 years (73.2%) after their diagnosis (*p* = 0.003) and those 2–5 years (72.8%) after their diagnosis (*p* = 0.003) (Fig. 1a).

The proportion of peer support needs to unmet needs tended to increase with cancer stage. The need for peer support in patients with stage 1 and stage 4 cancer showed a significant difference in the social/religious/spiritual support domain (55.9 vs. 84.4%, *p* = 0.005), as well as in practical support domain (60 vs. 81.8%, *p* = 0.04) (Fig. 1b).

The proportion of peer support needs to unmet needs varied by cancer type. Peer support needs in the information domain were significantly higher for stomach cancer (96.6%) than for breast (79.1%, *p* = 0.03), colorectal (67.3%, *p* = 0.002), and prostate cancer (71.4%, *p* = 0.02). In the social/religious/spiritual support domain, peer support needs of breast cancer (78.4%) were significantly different to colorectal (73.9%, *p* = 0.009), lung (46.2%, *p* = 0.01), thyroid (59%, *p* = 0.02), and prostate cancer (50%, *p* = 0.02) (Fig. 1c).

## Discussion

To the best of our knowledge, this is the first study to evaluate the need for peer support in managing the unmet needs of cancer patients according to stage in cancer trajectory. Our study revealed that two thirds of the patients who reported unmet needs in comprehensive cancer care also said that they wanted peer support in information-related needs, psychological care, and physical symptoms. The longer patients had been diagnosed with cancer and the higher their stage at cancer diagnosis was, the higher their demand was for obtaining information on cancer through peer support. Additionally, patients diagnosed with advanced cancer needed peer support in the social/religious/spiritual and practical domains.

Peer support needs in the information domain were consistently high throughout all periods of patients’ cancer journeys, indicating that cancer patients still wished to be well informed through peer support. Patients with more than 5 years since their cancer diagnosis, who were in the disease-free status, seemed to need more information about their health through peer support. They seldom meet their doctors or nurses after completing treatment, despite continuing to have health problems such as fear of recurrence and discomfort with being associated with the disease. In a review article, long-term breast cancer survivors were found to have ongoing informational and emotional needs, but they did not consistently receive help from medical staff [6]. This lack of medical support may lead them to look for complementary sources of help such as peer support.

In addition, advanced cancer patients showed a higher need for peer support in the social/religious/spiritual support and practical domains. In the literature, a review of unmet needs of patients with advanced cancer showed that unmet needs were most prevalent in the informational and psychological/psychosocial domains in that order [5]. Coping with advanced cancer means having to live with uncertainties and doubts. Moreover, as cancer progresses, there may be more economic problem or conflict between partners, and family members or friends. In this case, peers can provide advice on relationships to patients based on their own experiences. Peer support seems to have no beneficial effects on survival of advanced cancer patients; however, it has been shown to improve psychological outcomes of them [22].

Compared with other domains, patients showed a lower need for peer support in social/religious/spiritual domains. This finding might be due to Korean cultural traits of refraining from talking about private matters with others who are not familiar with the situations. As harmony in interpersonal relationships is highly valued, people in Asian cultures tend to suppress expression of negative emotions, and their response to illness is often serenity [23]. Patients rely on people who are close to them and who can help them, and friends or family gatherings are more essential in some aspects to satisfying their needs. Among Japanese cancer patients, peer support for social-spiritual needs is sought less than support from family and friends [13]. These patients gained spiritual or social support from these closer relationships.

However, breast cancer patients needed more peer support for social/religious/spiritual issues than patients with other types of cancer. This finding can be attributed to the characteristics of breast cancer in Korea, where the prevalence of premenopausal breast cancer and the survival rate are higher than in Western countries. The incidence of breast cancer among people in their 40s is highest in Korea and is 15% in patients under 40 years of age [24]. The 5-year survival rate of breast cancer in Korea is 91.5% (2008–2013) [25], which is higher than the rates of 89.8% in the USA (2006–2012) [26].

Peer support programs are delivered in different formats and in numerous contexts. In a systematic review, five types of peer support were identified: one-on-one telephone, one-on-one face-to-face, group telephone, group face-to-face, and group internet support [17]. In several interventional studies, peers developed skills in listening and providing empathy, strengthened their self-care strategies under supervision from a nurse [27], and were trained to mentor through lectures, group discussion, or role play [28]. In peer counseling sessions, they received detailed treatment manuals for educating cancer patients [29]. Therefore, it is essential to consider cancer patients’ specific needs and to deliver interventions according to sociodemographic and clinical factors when designing and implementing mentor programs for cancer patients.

Our study has several limitations. We focused on peer support to be concerned with cancer patients’ unmet needs. Participants were not given the opportunity to indicate other types of support such as friends, family, or non-medical professors that might be helpful. However, in this study, we wanted to assess cancer survivors’ role as mentors who would share their experiences with and give advices to cancer patients. Further research is needed to examine the needs of other types of support, and the effectiveness of peer support programs in fulfilling these patients’ unmet needs as well. Second, peer support needs were examined using a tool derived from a validated instrument that assessed comprehensive cancer care. This tool for peer support has not been validated; however, it showed high internal consistency in this study. Finally, this study was conducted in a local hospital, and the distribution of sex and cancer type differed from that of national data. Prevalence of cancer according to the national cancer statistics were as follows: 22.0% for thyroid cancer, 15.9% for stomach cancer, 13.7% for colorectal cancer, 11.1% for breast cancer, and 4.3% for lung cancer [30]. In our study population, breast cancer accounted for one third of our study population, while thyroid cancer and stomach cancer was under-sampled compared with national data. Therefore, the study population was limited in its ability to represent the general population and may have introduced selection bias. Sociodemographic factors such as age, sex, marital status, and income level as well as clinical factors such as location of cancer, stage of disease, and tumor size can influence unmet needs in cancer patients [31]. In this regards, our study might not represent the unmet needs in male, elderly cancer survivors, and gastric cancer or thyroid cancer survivors. Future studies among representative cancer patient groups are needed.

Despite these limitations, our study results suggest that cancer patients have various unmet needs, and also need for peer support. To help them at the right time, it is important to evaluate their needs in detail and provide peer support programs based on their specific needs.

## Electronic Supplementary Material

ESM 1 (DOCX 20 kb)
