# Supplementary material for: Could Peer Support Programs Be a Good Resource for Managing the Unmet Needs of Cancer Patients?
Source: J Cancer Educ. 2018 Aug 8;34(5):950–7. doi: 10.1007/s13187-018-1399-4 (PMC6785582; doi:10.1007/s13187-018-1399-4)
Supplement: Supplementary file 1 — (DOCX 20 kb) [file 13187_2018_1399_MOESM1_ESM.docx]

| Appendix 1 Needs of peer group support in patients with cancer | |
| --- | --- |
| Domain | Items |
| Information | Needed peer support to get an information about current status of my illness and its future courses |
|  | Needed peer support to get an information about tests and treatments |
|  | Needed peer support to get an information about symptoms require a hospital visit |
|  | Needed peer support to get an an easy and accurate explanation about benefits, side effects and application of current medication |
|  | Needed peer support to get an information or education about things that I can do at home for my health |
|  | Needed peer support to get guidelines or information about complementary and alternative medicine |
|  | Needed peer support to get an information about correct diet (food to eat, food to avoid) |
|  | Needed peer support to get an information about cancer treating hospitals or clinics and physicians |
|  | Needed peer support to get an information about financial support for medical expenses from government |
|  | Needed peer support to get an information about hospice service |
| Psychological problem | |
|  | Needed peer support with feelings of unidentifiable anxiety |
|  | Needed peer support in coping with fear of recurrence |
|  | Needed peer support with worries about treatment sequelae |
|  | Needed peer support with my concerns for the family |
|  | Needed peer support with worries that I would become a burden to others around me |
|  | Needed peer support for depression |
|  | Needed peer support with feelings of anger, irritability, or nervousness |
|  | Needed peer support with loneliness or feelings of isolation |
|  | Needed peer support with accepting role changes at home, at work and/or in society after cancer diagnosis |
|  | Needed peer support with acceptance of changes in my appearances due to cancer |
| Physical symptoms | |
|  | Needed peer support with pain |
|  | Needed peer support with lack of energy and/or fatigue |
|  | Needed peer support with trouble sleeping or oversleeping |
|  | Needed peer support with diarrhea or constipation |
|  | Needed peer support with nausea and/or vomiting |
|  | Needed peer support with lack of appetite |
|  | Needed peer support wth loss of hair |
|  | Needed peer support with shortness of breath |
|  | Needed peer support with my body feeling a sense of numbness and/or tingling |
|  | Needed peer support with feeling of fever and/or hot flashes |
|  | Needed peer support with decline in my ability to concentration or memorize |
|  | Needed peer support with changes in sexual life |
| Social/Religious/spiritual support | |
|  | Needed peer support with difficulties that arose in family relationships after cancer diagnosis |
|  | Needed peer support with difficulties that arose in interpersonal relationships after cancer diagnosis |
|  | Needed peer support to religious support |
|  | Needed peer support in finding the meaning of my situation and in coming to terms with it |
| Practical support |  |
|  | Needed peer support to use transportation services for getting to and from the hospital |
|  | Needed peer support with my economic burden due to cancer |
|  | Needed peer support to seek someone to help me with housekeeping and/or child care |
|  | Needed peer support to have assisted care in the hospital or at home |

| Appendix 2 Top 3 items of peer support needs in the social/religious/spiritual domain | | | | | | |
| --- | --- | --- | --- | --- | --- | --- |
|  | Rank | | | | | |
|  | 1 (item, %) | | 2 (item, %) | | 3 (item, %) | |
| Cancer type |  |  |  |  |  |  |
| Breast | B | 40.5 | A | 39.9 | C | 38.5 |
| Colon/Rectum | B | 33.3 | D | 29.3 | A | 28.0 |
| Lung^a^ | - |  | - |  | - |  |
| Thyroid | C, D | 40.9 | B | 36.4 | A | 29.5 |
| Stomach | D | 50.0 | B | 47.2 | C | 38.9 |
| Prostate | C | 36.4 | A, B | 27.3 | D | 22.7 |
| Others^b^ | C | 43.1 | A | 41.4 | B | 39.7 |
| Duration |  |  |  |  |  |  |
| <3months | B | 45.6 | D | 42.6 | A | 41.2 |
| 3months-2years | B | 35.6 | A | 33.6 | C | 31.5 |
| 2-5 years | B, C | 37.2 | D | 35.4 | A | 32.7 |
| ≥5years | C | 34.7 | B | 34.7 | A | 33.3 |
| Cancer stage |  |  |  |  |  |  |
| 1 | B | 30.0 | A | 29.2 | C | 28.3 |
| 2 | C | 38.3 | D | 37.6 | A, B | 36.2 |
| 3 | B | 36.2 | C | 37.1 | D | 37.1 |
| 4 | B, D | 50.0 | C | 47.7 | A | 43.2 |
| Item A : Needed peer support with difficulties that arose in family relationships after cancer diagnosis Item B : Needed peer support with difficulties that arose in interpersonal relationships after cancer diagnosis Item C : Needed peer support to religious support Item D : Needed peer support in finding the meaning of my situation and in coming to terms with it ^a^All four items of the domain showed same preference in lung cancer.  ^b^Included esophagus, kidney, liver, pancreas, prostate, ovary, cervix, leukemia and lymphoma | | | | | | |
